# Comparison of Cancer Cell Elasticity by Cell Type

**DOI:** 10.7150/jca.45897

**Published:** 2020-07-11

**Authors:** Sangwoo Kwon, Woochul Yang, Donggerami Moon, Kyung Sook Kim

**Affiliations:** 1 Department of Biomedical Engineering, College of Medicine, Kyung Hee University, Seoul 130-710, Republic of Korea.; 2 Department of Physics, Dongguk University, Seoul 04620, Republic of Korea.

**Keywords:** Cellular elasticity, breast cancer, cervix cancer, lung cancer, atomic force microscopy, F-actin

## Abstract

Lower cellular elasticity is a distinguishing feature of cancer cells compared with normal cells. To determine whether cellular elasticity differs based on cancer cell type, cells were selected from three different cancer types including breast, cervix, and lung. For each cancer type, one counterpart normal cell and three types of cancer cells were selected, and their elasticity was measured using atomic force microscopy (AFM). The elasticity of normal cells was in the order of MCF10A > WI-38 ≥ Ect1/E6E7 which corresponds to the counterpart normal breast, lung, and cervical cancer cells, respectively. All cancer cells exhibited lower elasticity than their counterpart normal cells. Compared with the counterpart normal cells, the difference in cellular elasticity was the greatest in cervical cancer cells, followed by lung and breast cancer cells. This result indicates lower elasticity is a unique property of cancer cells; however, the reduction in elasticity may depend on the histological origin of the cells. The F-actin cytoskeleton of cancer cells was different in structure and content from normal cells. The F-actin is mainly distributed at the periphery of cancer cells and its content was mostly lower than that seen in normal cells.

## Introduction

Cancer cells show unique characteristics including undifferentiated states, abnormal nuclei, lack of normal signaling pathways, abnormal energy metabolism and vascularization 1-3. Compared with healthy normal cells, cancer cells also exhibit the unique physical property of elasticity. Cellular elasticity indicates the ability of the cell to deform in response to external stress. Recently, cancer cells were shown more elastic than normal cells 4,5. Metastatic cancer cells taken from the body fluids of patients with suspected lung, breast, and pancreatic cancer were over 70% more elastic than benign cells 4.

Differences in the cellular elasticity of cancer cells may be a characteristic of carcinogenesis or may be part of a survival strategy to adapt to new environments. Carcinogenesis is the process by which normal, healthy cells transform into cancer cells. Carcinogenesis proceeds in multiple steps: initiation, promotion, and progression over a period of several decades 6. Carcinogenesis is caused by DNA mutations that alter the normal balance between cell proliferation and cell death 7. The chemical or structural changes that occur during carcinogenesis may result in low elastic property of cancer cells. Carcinogenesis affects not only the cells but also the surrounding extracellular matrix (ECM) 8. Highly proliferative cancer cells vigorously secrete growth factors that stimulate fibroblasts, key regulators of ECM composition and organization. Activated fibroblasts overproduce ECM proteins such as collagen I and fibronectin, which may result in ECM stiffening. The mechanical stress exerted by a stiff ECM can regulate cancer cell properties including elasticity, morphology, growth, and motility 9,10.

Although the underlying mechanism remains unclear, the lowering in cellular elasticity occurs in most cancer types. Therefore, the elastic property of cancer cells has been considered a biomarker for early diagnosis or metastatic potential 11. However, the degree of alteration in cellular elasticity may differ based on cancer cell type. We reviewed the relationship between lowered elasticity and cancer type in previous work 12. The difference in elasticity between normal and cancer cells was compared in eight types of cancer cells, including lung, kidney, prostate, breast, thyroid, bladder, ovarian, and esophageal cells. Compared with normal cells, bladder cancer cells showed significantly low Young's modulus in elasticity (more than 80%), however, the elasticity of esophageal cancer cells was relatively low Young's modulus (approximately 40%). The difference in elasticity between breast and thyroid cancer cells ranged from 20-80%. The difference in elasticity depending on cancer cell type may reflect characteristics of the cancer cell, such as mobility and metastasis, or may result from a difference in cellular elasticity measurement conditions. Cellular elasticity is generally measured using atomic force microscopy (AFM). Since the measured elasticity of cells depends on AFM measurement conditions such as probe shape and loading rate, comparisons of the results obtained under different conditions may have a certain degree of error 13.

In the present study, cellular elasticity of various cancer cells under the same conditions was measured to eliminate any measurement errors. Three different cell types including breast, cervix, and lung cancers, were selected. One counterpart normal cell and three cancer cells were obtained from each type. Among the cancer cells, two were non-metastatic cells and one was a metastatic cell. Cellular elasticity measurements were performed using AFM in liquid conditions. Differences in the content and distribution of F-actin, a main structural protein in the cell cytoskeleton, were examined, and the results compared with cellular elasticity.

## Materials and Methods

### Cell culture

Three different types of cancer cells, breast (MCF7, T47D, MDA-MB-231), cervix (HeLa, SiHa, Caski), and lung (A549, H460, H1299) were purchased from KCLB (Korean Cell Line Bank, Seoul, Korea). Counterpart normal cells MCF10A, Ect1/E6E7, and WI-38 for breast, cervix, and lung cancers, respectively, were purchased from ATCC (ATCC Inc., Manassas, VA, USA). The culture medium for MCF10A included Mammary Epithelial Cell Basal Medium (MEBM) (Lonza Inc., Basel, Switzerland), 0.4% bovine pituitary extract (BPE), 0.1% human epidermal growth factor (hEGF), 0.1% hydrocortisone, 0.1% insulin, and 100 ng/mL cholera toxin. MCF7, T47D, MDA-MB-231, Caski, A549, H460, and H1299 cells were cultured using Roswell Park Memorial Institute (RPMI) 1640 Medium (Thermo Fisher Scientific, Waltham, MA, USA) with 10% FBS, 300 mg/L L-glutamine, 25 mM hydroxyethyl piperazineethanesulfonic acid (HEPES), and 25 mM NaHCO_3_. Ect1/E6E7 cells were cultured in Keratinocyte Serum-Free Medium (SFM) with 0.05 mg/mL BPE, 0.1 ng/mL epidermal growth factor (EGF) and 44.1 mg/L calcium chloride. Minimum Essential Medium (MEM) (Thermo Fisher Scientific) with 10% FBS and DMEM with 10% FBS were used to culture HeLa and SiHa cells, respectively. Eagle's Minimum Essential Medium (EMEM) was used to culture WI-38 cells. All cells were cultured in 37 °C and 5% CO_2_ conditions.

### AFM measurement

The AFM system Nano N8 NEOS AFM (BRUKER®, Hamburg, Germany) was used for imaging and force-distance (FD) curve measurement in liquid conditions. The AFM probe (Cont GD, Budget Sensors Inc., Sofia, Bulgaria) consisted of an Au-coated cantilever and a Si-based conical tip was used to measure the FD curve. Detailed dimensions of the probe were as follows: resonance frequency, 13 kHz (± 4 kHz); force constant, 0.2 N/m (0.07-0.4 N/m); cantilever length, 450 µm (± 10 µm); cantilever width, 50 µm (± 5 µm); cantilever thickness, 2 µm (± 1 µm); tip height, 17 µm (± 2 µm); tip radius < 10 nm. Half cone angle of the conical tip along the cantilever-axis ranged from 20-22.5°. Load force was set at ≤ 10 nN to minimize damage to the cell membrane, and the loading rate of the probe was approximately 1 µm/s. FD curve measurement was performed at 10 points per cell for 20 cells of each cell type. The cellular elasticity was measured around the cell nucleus to avoid matrix effects. For FD curve measurement, cells were immobilized with 3.7% formaldehyde solution for 15 min.

### Immunofluorescence staining

All cancer cells were treated with 3.7% formaldehyde solution for 15 min to fix the cell structure. Cells were then washed with phosphate buffer saline (PBS) solution for 30 s. Rhodamine-phalloidin (100 nM, Alexa Fluor 488 phalloidin, Invitrogen Inc., Carlsbad, CA, USA) was used to react stoichiometrically with F-actin for visualizing distribution of actin filaments. Reagent-treated cells were incubated for 30 min at room temperature and in dark conditions. Cells were rewashed several times with PBS and stored in dark conditions at 4 °C. Fluorescence optical microscope (NIKON Ti-E, Nikon instruments Inc., Tokyo, Japan) was operated using an excitation wavelength of approximately 495 nm and an emission wavelength of approximately 518 nm. Immediately prior to fluorescence measurement, 4',6-diamidino-2-phenylindole, dihydrochloride (DAPI) solution was used to stain nuclei. DAPI has absorption and emission maxima at wavelengths of 358 nm (ultraviolet) and 461 nm (blue), respectively.

### Western blotting

Cells were washed several times with PBS and scraped into RIPA buffer containing a protease inhibitor cocktail. To separate cell debris and actin proteins, cells were centrifuged at 374 ×G for 5 min (4 °C). Then, the supernatant was continuously centrifuged at 15,000 ×G for 5 min (4 °C). The pellet that formed on the bottom contained F-actin and the supernatant contained G-actin. For western blot analysis, F-actin was treated with depolymerization buffer solution to transfer G-actin. Then, 10 mg of G- or F-actin proteins were loaded onto 12.5% polyacrylamide gels; the resolved proteins were transferred to nitrocellulose membranes. The membranes were blocked with 5% fat-free milk in PBS (pH 7.4) for 30 min at room temperature and then incubated with anti-actin (Cytoskeleton Inc., Denver, CO, USA) at the appropriate dilutions overnight at 4 °C. Membranes were incubated with anti-rabbit secondary antibodies (GenDEPOT Inc., Katy, CO, USA) for 1 h at room temperature and then subjected to enhanced chemiluminescence (Pierce Biotechnology, Waltham, MA, USA) and autoradiography using the ChemiDoc XRS+ Imaging System (BioRad, Hercules, CA, USA).

## Results

### Morphological characteristics of cancer cells based on cancer type

**Figure 1:**
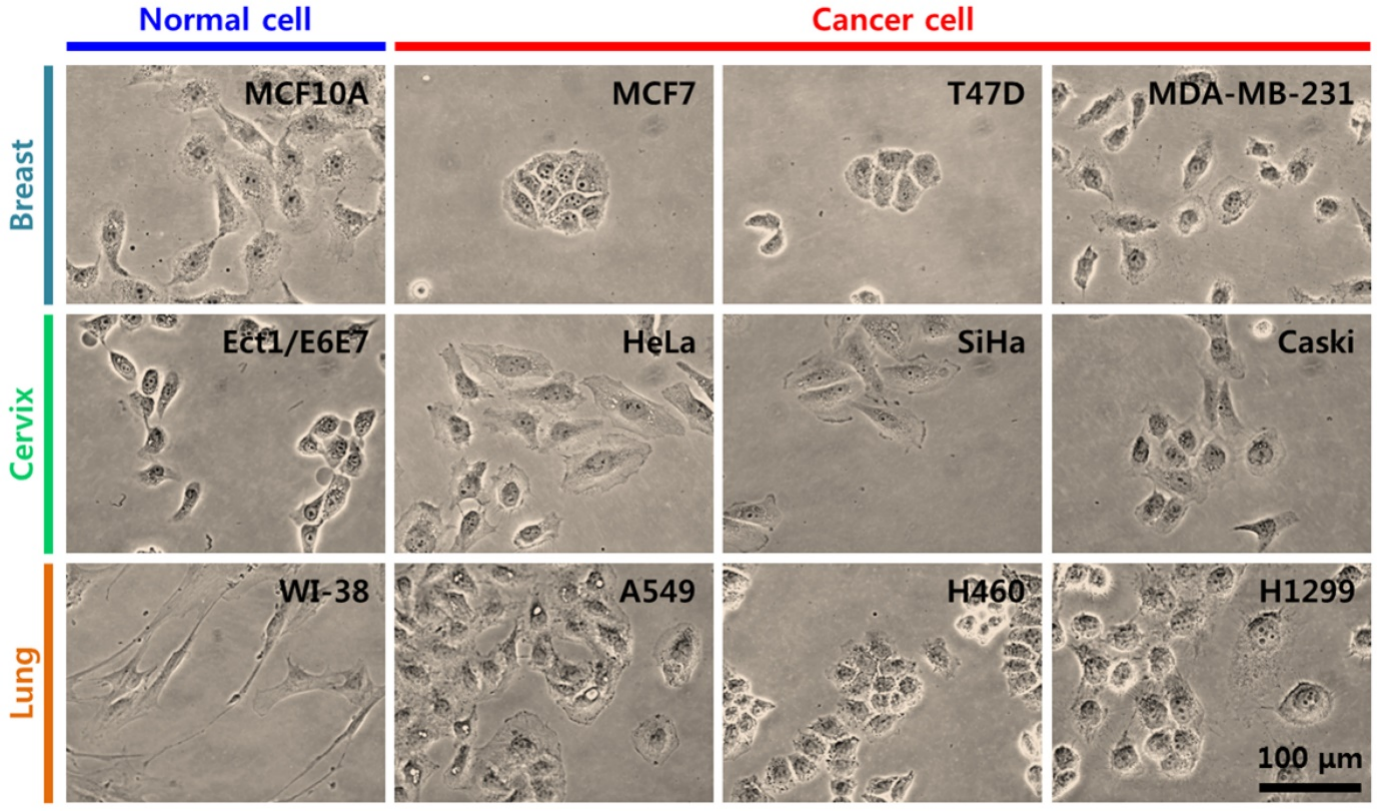
Optical images both normal cell and three kinds of different cancer cells within different types of tumour such as breast, cervix, and lung.

Breast, cervical, and lung cancer cells were used in the present study (Table 1) 14-18. For breast cancer, MCF10A cells were selected as counterpart normal cells, and MCF7, T47D, and MDA-MB-231 cells were selected as cancer cells. For cervical cancer, Ect1/E6E7 cells were the counterpart normal cells, and HeLa, SiHa, and Caski cells were cancer cells. For lung cancer, WI-38 cells were selected as counterpart normal cells, and A549, H460, and H1299 cells were selected as cancer cells. For each group of cancer cells, two cell lines were non-metastatic and one was metastatic. MDA-MB-231, Caski, and H1299 cells are metastatic cells. The morphology and growth pattern of cells were observed using an optical microscope (magnification ×400). Figure 1 shows the optical microscopy images of cancer cells and their counterpart normal cells. In breast cancer cells, most MCF10A normal and MDA-MB-231 cancer cells were observed as a single cell state, however, MCF7 and T47D cancer cells grew in a cluster. In cervical cancer cells, both normal (Ect1/E6E7) and cancer cells grew individually with particular spacing without clustering. In lung cancer cells, WI-38 normal and A549 and H1299 cancer cells grew independently, however, H460 cancer cells created a cell colony.

### Cellular elasticity measurements and elasticity comparison between cancer cells and counterpart normal cells

**Figure 2:**
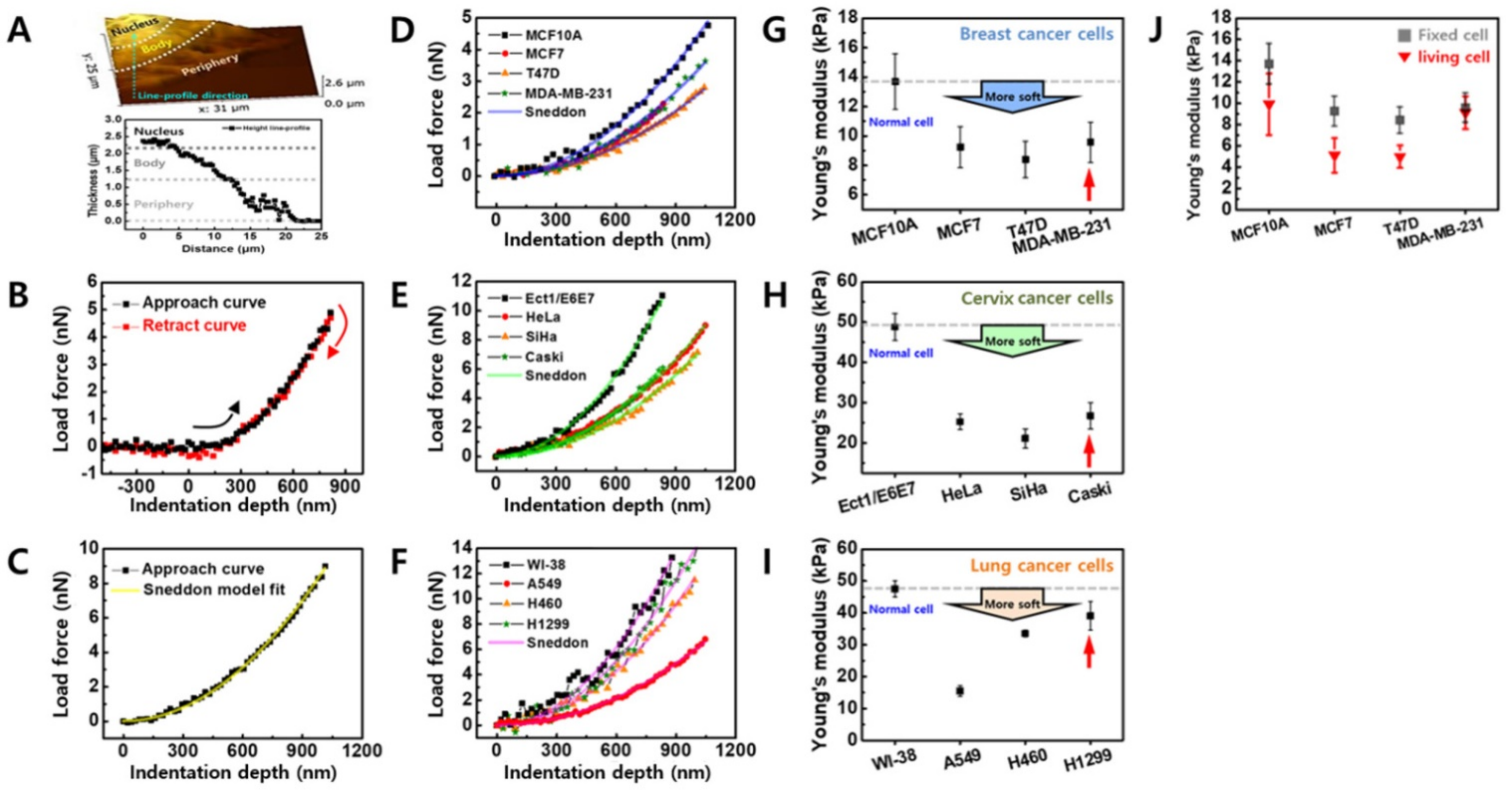
(**A**) Representative AFM images of cells. Colors indicate different heights. Light and dark colors correspond to higher and lower topography, respectively. The nucleus, body, and periphery sites are marked in height line-profile. (**B**) Representative force-distance curve. Black rectangle and red rectangle correspond to approach and retract processes, respectively. (**C**) Young's modulus was calculated from approach curve using Sneddon model. (**D-F**) Typical FD curves obtained from cells selected from breast, cervix, and lung tissues and their fitted results using a Sneddon model, respectively. (**G-I**) Determined Young's modulus of cells from breast, cervix, and lung tissues, respectively. (**J**) Comparison of Young's modulus between normal cell and cancer cells for breast cells in both fixed and living conditions.

The cellular elasticity was determined based on FD curve measurement using AFM. After imaging a cell surface with a scan 20 × 20 μm^2^ in size, the actual contact point for the FD curve was selected for the cell body (Figure 2A). Although cell thickness (the difference in height between the cell nucleus and the substrate) varies by cell, the range is approximately 2.5 µm, and becomes thinner as it nears the periphery. The cell thickness at the periphery is approximately 1 µm. Based on this data, the FD curve was obtained for the cell body around the nucleus due to the following two reasons. First, the cytoskeletal proteins that contribute to cellular elasticity are abundant around the nucleus. Second, if the cell membrane is very thin (< 2 µm); the FD curve reflects the influence of the substrate.

The FD curves of individual cells were extracted using the magnitude of loading force on the sample based on the indentation depth of the cell (Figure 2B). Depending on conditions such as AFM tip shape, Hertz or Sneddon model was used to calculate Young's modulus from the FD curve 19. In the present study, a Sneddon model was used to analyze the FD curve by considering a relatively sharp probe with a radius of approximately 10 nm. The Sneddon model is as follows 20:

*F* = *E* × {2 tan *α* / π × (1-*ν*^2^)} × *δ*^2^ (1)

where *F* and *δ* are measured values indicating load force and indentation depth, respectively, *α* is a half cone angle along the cantilever axis, and *ν* is Poisson's ratio. The *α* and *ν* values were fixed at 22.5° and 0.5, respectively. *E* is Young's modulus, a physical quantity of sample elasticity. FD curve fitting based on the Sneddon model was interpreted as having a high fitting ratio close to R^2^ > 0.99 for all cells, as shown in Figure 2C. A high Young's modulus value indicates high elasticity and a low value indicates low elasticity.

Figure 2D shows FD curves measured in the counterpart normal cells (MCF10A) and breast cancer cells (MCF7, T47D, and MDA-MB-231) and a clear difference in elasticity was observed between the cells. The Young's modulus of breast cancer cells was approximately 30-40% lower compared with the counterpart normal cells (Figure 2G and Table 2). The difference in cellular elasticity between normal and cancer cells was more apparent in cervical cancer cells (Figures 2E and 2H). The counterpart normal cells showed a large Young's modulus of 48.77 ± 3.33 kPa; however, the values of cancer cells ranged from 21.09-26.73 kPa (Table 2). The reduced rate of Young's modulus in cancer cells was approximately 45-57% compared with normal cells. Although lung cancer cells were softer than normal lung cells, differences in the Young's modulus of cancer cells were widely distributed (Figures 2F and 2I). Compared with normal cells (WI-38), A549 was 67% softer, H460 was 29% softer, and H1299 was only 18% softer (Table 2). Notably, metastatic cancer cells exhibited higher elasticity than non-metastatic cells in all groups. In breast cancer cells, MDA-MB-231 had higher Young's modulus than MCF7 and T47D. In cervical and lung cancer cell groups, the Young's modulus of metastatic cells (Caski and H1299) was higher than non-metastatic cells.

Due to the difficulties in applying AFM to living cells, the cellular elasticity in all groups was determined using fixed cells which were treated with 3.7% formaldehyde solution for 15 min. Because formaldehyde fixes the cells by cross-linking the proteins, the fixed cells exhibit different elastic properties than living cells. Therefore, to assess the elasticity based on cancer type, FD curves were also measured in living cells under the same conditions used for fixed cells (Figure 2J). The Young's modulus of living cells was 9.8 ± 2.89 kPa (MCF10A), 5.0 ± 1.62 kPa (MCF7), 4.9 ± 1.07 kPa (T47D), and 9.0 ± 1.53 kPa (MDA-MB-231). Thus, the living cells were approximately 28-45% less elastic than fixed breast cancer cells, except for the living MDA-MB-231 cells which showed almost similar elasticity to the fixed cells. Although the Young's modulus of living cells was lower than fixed cells, the difference in elasticity was similar between the living cells and the fixed cells.

### Lower F-actin levels in cancer cells

Quantitative analysis of actin protein was performed to examine cytoskeletal differences in cancer cells. Actin protein is an essential component of the cytoskeleton and plays a major role in cellular elasticity 21. The actin protein has two forms, a globular monomer (G-actin) and a filamentous polymer (F-actin). F-actin is formed by polymerization from G-actin and is closely related to the elasticity of living cells. Since the total amount of G- and F-actin is maintained through the polymerizing process, the relative amount of F-actin was compared in cancer and normal cells.

**Figure 3:**
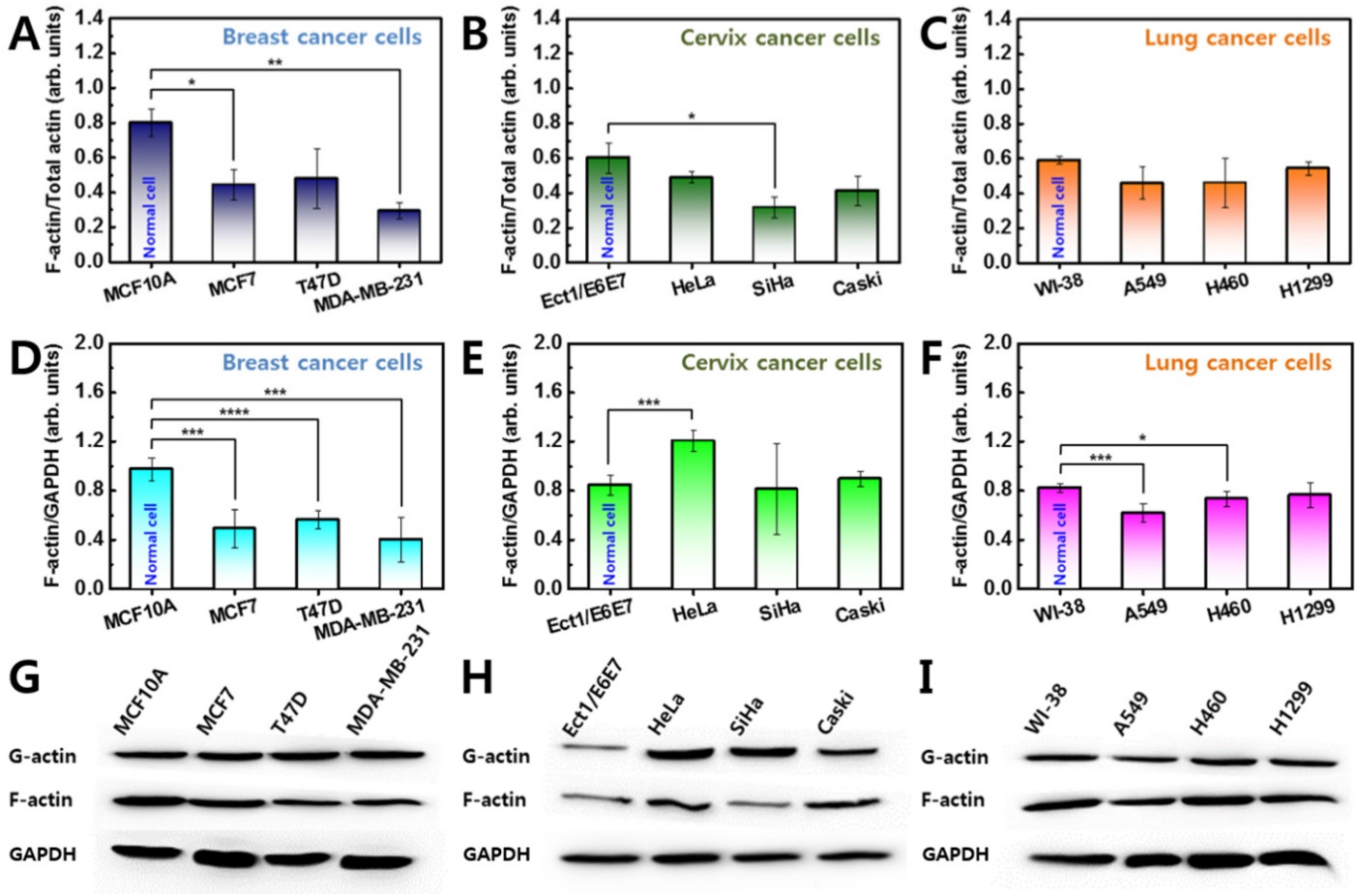
Western blot analysis of G-actin and F-actin was conducted for all cells from (**A**) breast, (**B**) cervix, and (**C**) lung tissues. Ratio of F-actin to total (G + F) actin was calculated in all cells, and ratio of normal cell was compared with that of cancer cells. (**D-F**) F-actin was also normalized by GAPDH. (**G-I**) The F-actin contents were compared with almost same amount of GAPDH. Error bars indicate ± SEM (standard error of the mean). *p ≤ 0.05; **p ≤ 0.01; ***p ≤ 0.001; ****p ≤ 0.0001 compared to the control.

Significant differences in F-actin content were observed in all breast cancer cells compared with the counterpart normal cells (Figure 3A). The measurements were repeated for three different batches of cells. The relative proportion of F-actin content was approximately 0.80 ± 0.08 in MCF10A, however, the content was lower in cancer cells ranging from 0.29 ± 0.05-0.48 ± 0.17. This result indicates that breast cancer cells have approximately 40-66% less F-actin than their counterpart normal cells. Differences in F-actin content were also confirmed in cervical and lung cancer cells (Figures 3B and 3C). In cervical cells, the relative F-actin content was 0.60 ± 0.08 in normal cells (Ect1/E6E7), however, the content ranged from 0.31 ± 0.05-0.41 ± 0.08 in cancer cells (HeLa, SiHa, and Caski) (Figure 3B). In lung cells, the relative content of F-actin was 0.59 ± 0.02 in normal cells (WI-38) and ranged from 0.46 ± 0.09-0.54 ± 0.04 in lung cancer cells (A549, H460, and H1299) (Figure 3C). Cervical and lung cancer cells have 34-50% and 9-22% less F-actin content, respectively, compared with their counterpart normal cells. On average, the relative proportion of F-actin content in normal cells was higher than in cancer cells regardless of the cell type (S1 Figure).

GAPDH was used as a standard to confirm the F-actin content was normalized in all groups (Figures 3D-I). In breast and lung cancer cells, F-actin content normalized with GAPDH was similar to the relative proportion of F-actin as shown in Figures 3A and 3B. However, cervical cancer cells showed different results (Figure 3E). The normalized F-actin content in HeLa cells was significantly greater than in Ect1/E6E7 cells which was probably because the HeLa cells are the largest in the cervical cancer group. Assuming the F-actin content is proportional to the cell size, the actin content was divided by the cell size. The divided F-actin contents showed similar results as shown in Figure 3B.

### Structural differences of F-actin in cancer cells

**Figure 4:**
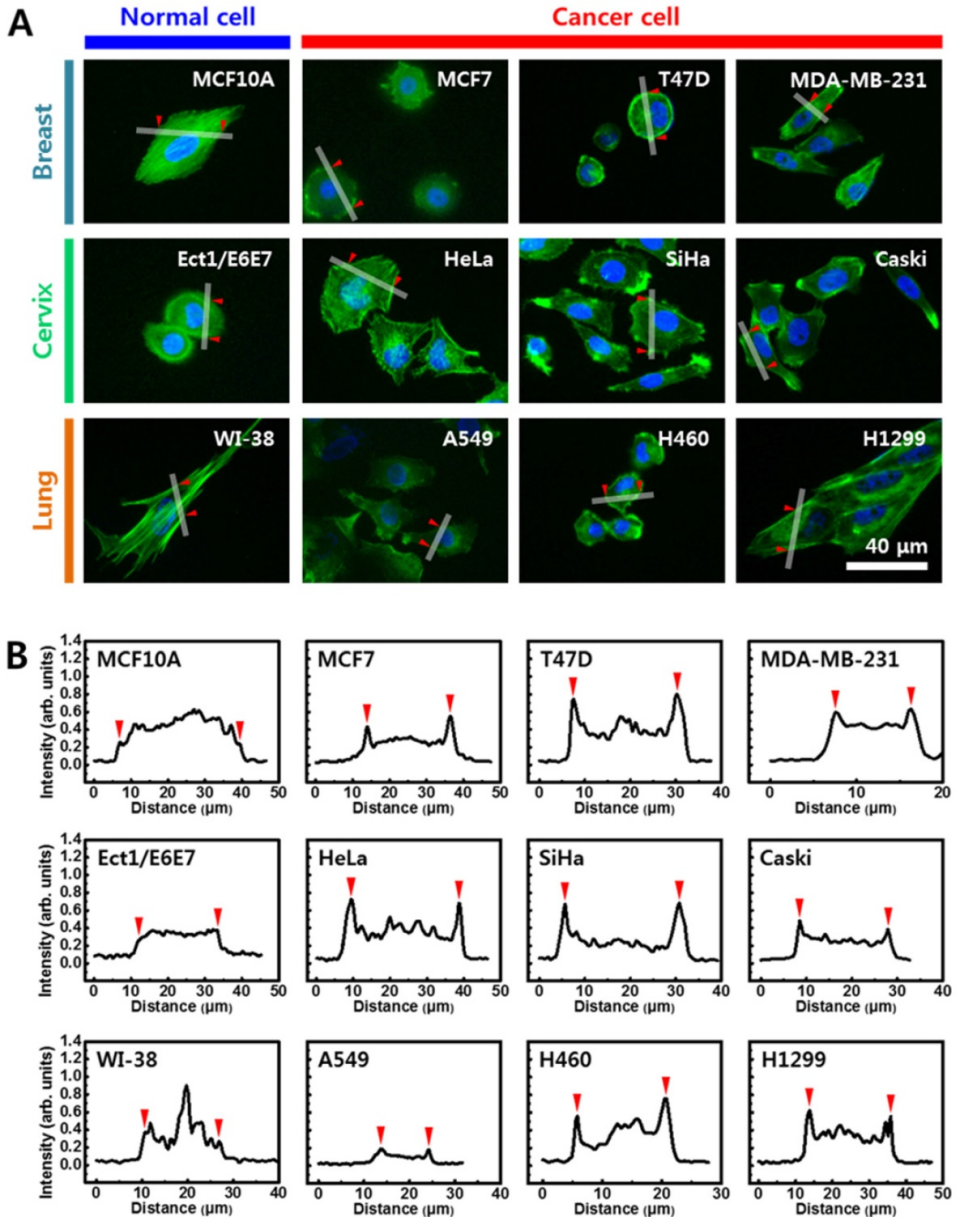
(**A**) Cells were stained with rhodamine-conjugated phalloidin to detect F-actin (green). (**B**) Quantification of fluorescence intensity of F-actin using Gwyddion software. Intensity profiles were determined from white line shown in (A). Red triangles indicate start and end points of line profiles.

Since cellular elasticity is also affected by cytoskeletal structure, the distribution of F-actin in cytoplasm was investigated using immunofluorescence images stained with rhodamine conjugated to phalloidin. Figure 4A shows fluorescence images of the F-actin in cells from breast (upper panel), cervical (middle panel), and lung (lower panel) cells. Distinct differences in F-actin distribution were observed between normal and cancer cells. In cells from breast, F-actin was evenly distributed in the normal cells (MCF10A) throughout the cytoplasm, however, F-actin was biased towards the periphery in cancer cells (MCF7, T47D, and MDA-MB-231). Different F-actin distribution in normal and cancer cells was more apparent in cells from cervix and lung. Unlike the uniform distribution of the normal cells, F-actin in the cancer cells was mainly distributed on the periphery. The density of F-actin was analyzed based on quantification of fluorescence intensity using Gwyddion software (Figure 4B). Fluorescence intensity was measured across the lines shown in the corresponding image in Figure 4A (S3 Figure). Significant difference was not observed in the intensity of F-actin across the cytoplasm in the three normal cell lines. However, the intensity of F-actin was significantly high at both peripheral regions in all cancer cells. The intensity of F-actin at the peripheral regions was more than twice the actin levels in other regions.

## Discussion

Carcinoma is a cancerous tumor that starts in cells of the epithelial tissue lining organs such as the liver, lung, breast, or kidneys 22. Like other types of cancer, carcinomas can divide without control and migrate to other parts of the body. Carcinoma is broadly divided into two main groups, squamous cell carcinoma and adenocarcinoma depending on where they originate. Squamous cell carcinomas arise from epithelial cells forming a protective cell layer that is thin and flat 23. Squamous cells are located in the anus, cervix, head and neck, and vagina. Adenocarcinomas originate in glandular cells, which are found in the glandular tissue of organs such as the cervix, lung, colon, and uterus 24. In the present study, cervical cancer cells were from squamous cell carcinomas and breast cancer cells were from adenocarcinomas 18. Lung cancer cells were from both squamous cell carcinomas and adenocarcinomas 14,15.

Cancer cells are characterized not only by their biological properties but also their physical properties. In several studies, cancer cells were reportedly softer than normal cells 3,4. As mentioned earlier, bladder cancer cells are 80% softer and esophageal cancer cells are 40% softer than normal cells 12. However, why cancer cells are softer than normal cells or how much softer they are remains unclear. In the present study, cancer cells originating from breast, cervix, and lung tissues were softer than their counterpart normal cells. However, the elasticity of cancer cells was dependent on the cell type. On average, breast cancer cells were 34% softer, lung cancer cells were 40% softer, and cervical cancer cells were 50% softer than their counterpart normal cells. Since these three different cancer cells have biologically similar epithelial origins, knowing whether the difference in elasticity is correlated with the biological origin of the cancer cells is difficult. Considering only the biomechanical properties of cells, softening of cancer cells appears to correlate with the elasticity of normal cells. When comparing only normal cells, breast cells (MCF-10A) were the softest, followed by lung (WI-38), and cervical (Ect1/E6E7) cells. The difference in elasticity between cancer cells and normal cells was the largest in cells from the cervix, followed by lung and breast tissues. This result indicates that differences in elasticity are greater when relatively rigid normal cells become cancer cells.

The F-actin cytoskeleton plays a major role in cellular elasticity. Cellular elasticity generally increases as F-actin content increases and decreases with decreased F-actin content 25. The F-actin content was lower than normal cells in all studied cancer cells than normal cells. However, the reduction rate of F-actin showed no correlation with changes in elasticity. The F-actin contents was 49.35% lower in breast cancer cells, in which the elasticity reduction rate was the smallest, and F-actin contents was 32.47% lower in cervical cancer cells, which showed the greatest reduction in cellular elasticity. Both the amount and distribution of F-actin had considerable effects on cellular elasticity. Structural differences of F-actin in cancer cells compared with normal cells are shown in Figure 4. However, the structural differences differed slightly based on cancer cell type. In some breast cancer cells, F-actin was distributed throughout the cytoplasm, although not as much as in normal cells. Therefore, differences in cellular elasticity may be high if F-actin is distributed throughout the entire cytoplasm, although the low contents of F-actin significantly. In both lung and cervical cancer cells, F-actin was mainly distributed at the periphery of the cytoplasm. Therefore, F-actin aggregation at the periphery may have a limited role in cellular elasticity.

**Figure 5:**
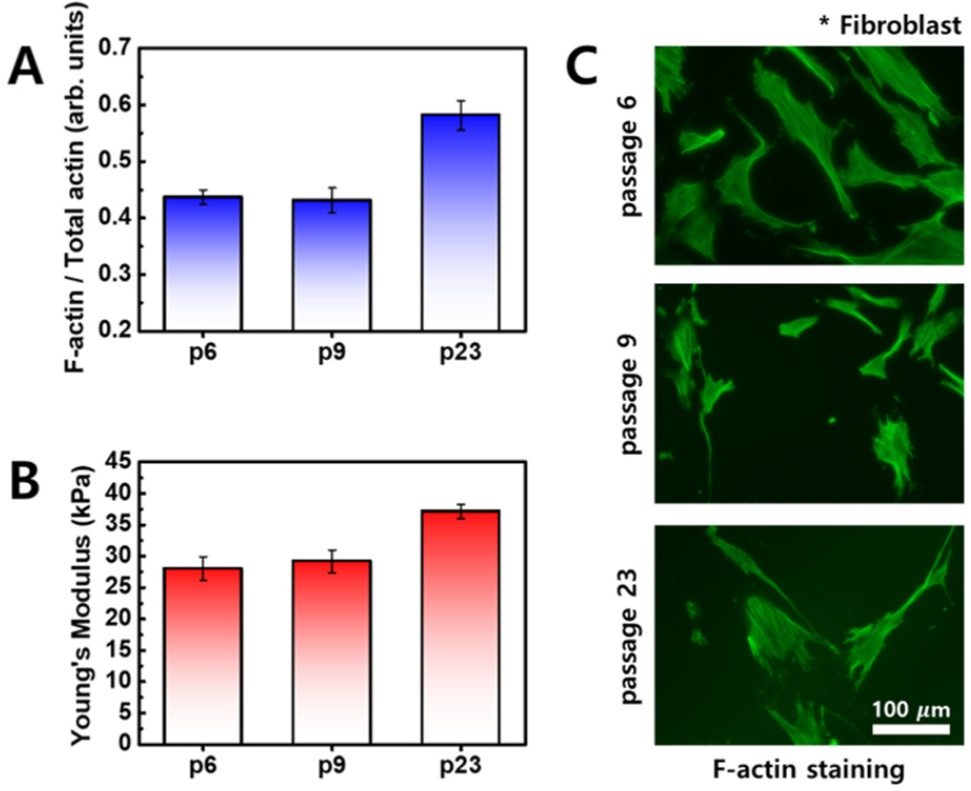
Fibroblasts were imaged using optical microscopy, and cells were stained with rhodamine-conjugated phalloidin to detect F-actin (green). (**A**) Ratio of F-actin to total actin as a function of cell passage. (**B**) Young's modulus of fibroblasts as a function of cell passages. (**C**) Fluorescence images of F-actin according to cell passages.

When comparing differences in cellular elasticity and F-actin between normal cells and cancer cells, normal cells also exhibited changes in elasticity that are mainly associated with age 26. As cell age increases, elasticity increases and F-actin content increases, as shown in Figure 5. Unlike cancer cells, distribution of F-actin was not significantly different in normal cells. Therefore, the difference in cellular elasticity based on the age of normal cells is likely due to F-actin content.

Metastatic cancer cells were less soft than non-metastatic cancer cells in all cancer types in the present study. Although the relationship between cellular elasticity and metastasis has not yet been elucidated, studies regarding the relationship between cellular elasticity and metastasis have indicated that elasticity is correlated with cell mobility which is directly linked to metastasis 11,27. Metastatic cancer cells were reportedly softer than non-metastatic cells. Ovarian cancer cells HEY and HEY A8 were softer than non-malignant ovarian surface epithelial cells, and the cancer cells showed greater invasive and migratory activity 11. Conversely, opposite results were recently published. According to Tae-Hyung Kim et al., metastatic breast cancer cells (MDA-MB-231) become less soft and more invasive with activation of β-adrenergic signaling 27. Our data presented in Figure 2 support that metastatic cancer cells (MDA-MB-231, Caski, and H1299) are less soft than non-metastatic cells.

In summary, although the elastic properties of cancer cells, which are distinguishable from normal cells, have been intensively studied, practical applications such as cancer diagnosis and treatment are limited. This is because the elasticity shows a very different value depending on the cell type as well as cell culture and measurement conditions, thus, quantitative analysis is limited. In the present study, more accurate analysis and comparison of cancer cell elasticity was possible by measuring the elasticity in three different groups having different histological origin under strictly controlled identical conditions. The elasticity of cancer cells was less compared with normal cells; however, the reduction in elasticity depended on the histological origin of the cancer cells. The change in cancer cell elasticity showed a close association with the change in actin content in all groups. In addition, the F-actin in the cancer cells was mainly distributed at the periphery of the membrane unlike normal cells. Further studies are needed to determine whether the unique distribution of F-actin in cancer cells is due to lower levels in actin content or associated with the mobility of cancer cells.

## Figures and Tables

**Table 1:** Tissues, cell line, cell type, morphologic, and histopathologic features of cells used in this study

Tissue	Cell line	Type	Morphology	Histopathology	ect.	Refs.
Breast	MCF-10A	Epithelial	Epithelial	None	Human breast epithelial cell line, mesenchymal-like appearance	14,15
	MCF7	Epithelial	Epithelial	Adenocarcinoma	More differentiated, noninvasive, tight cell-cell junction	14,15
	T47D	Epithelial	Epithelial	Ductal carcinoma	More differentiated, noninvasive	14,15
	MDA-MB-231	Epithelial	Epithelial	Adenocarcinoma	mesenchymal-like appearance, tight cell-cell junctions, metastatic	14,15
Cervix	Ect1/E6E7	Epithelial HPV-16 E6/E7 transformed	Epithelial	None	Human normal cervical cell line	16
	HeLa	Epithelial	Epithelial	Adenocarcinoma	HPV-18 genotype	17
	SiHa	Epithelial	Epithelial	Squamous cell carcinoma	HPV-16 genotype	17
	Caski	Epithelial	Epithelial	Squamous cell carcinoma	HPV-16 genotype, small bowel metastasis	17
Lung	WI-38	Fibroblast	Fibroblast	None	Fibroblast normal lung cell line	18
	A549	Epithelial	Epithelial-like	Adenocarcinoma	Low mobility	18
	H460	Epithelial	Epithelial	Carcinoma; large cell lung cancer	Large cell lung cancer	19
	H1299	Epithelial	Epithelial	Carcinoma	Non-small cell lung cancer, high mobility, metastatic	18

**Table 2:** Averaged Young's modulus of normal and cancer cells determined from FD curve

Group	Cell line	Young's modulus (kPa)	Relative value
Breast cancer	MCF-10A	13.69 ± 1.9	1.00
	MCF7	9.24 ± 1.39	0.68
	T47D	8.39 ± 1.24	0.61
	MDA-MB-231	9.57 ± 1.38	0.70
Cervical cancer	Ect1/E6E7	48.77 ± 3.33	1.00
	HeLa	25.25 ± 1.89	0.52
	SiHa	21.09 ± 2.42	0.43
	Caski	26.73 ± 3.23	0.55
Lung cancer	WI-38	47.52 ± 2.50	1.00
	A549	15.50 ± 1.74	0.33
	H460	33.54 ± 1.10	0.71
	H1299	39.04 ± 4.45	0.82
